# Silencing of *Anopheles stephensi* Heme Peroxidase HPX15 Activates Diverse Immune Pathways to Regulate the Growth of Midgut Bacteria

**DOI:** 10.3389/fmicb.2016.01351

**Published:** 2016-08-31

**Authors:** Mithilesh Kajla, Tania P. Choudhury, Parik Kakani, Kuldeep Gupta, Rini Dhawan, Lalita Gupta, Sanjeev Kumar

**Affiliations:** ^1^Molecular Parasitology and Vector Biology Laboratory, Department of Biological Sciences, Birla Institute of Technology and SciencePilani, India; ^2^Department of Zoology, Chaudhary Bansi Lal UniversityBhiwani, India; ^3^Department of Biotechnology, Chaudhary Bansi Lal UniversityBhiwani, India

**Keywords:** *Anopheles stephensi*, peroxidases, HPX15, mucin barrier, midgut bacteria, *Plasmodium*, innate immunity, vectorial capacity

## Abstract

*Anopheles* mosquito midgut harbors a diverse group of endogenous bacteria that grow extensively after the blood feeding and help in food digestion and nutrition in many ways. Although, the growth of endogenous bacteria is regulated by various factors, however, the robust antibacterial immune reactions are generally suppressed in this body compartment by a heme peroxidase HPX15 crosslinked mucins barrier. This barrier is formed on the luminal side of the midgut and blocks the direct interactions and recognition of bacteria or their elicitors by the immune reactive midgut epithelium. We hypothesized that in the absence of HPX15, an increased load of exogenous bacteria will enormously induce the mosquito midgut immunity and this situation in turn, can easily regulate mosquito-pathogen interactions. In this study, we found that the blood feeding induced AsHPX15 gene in *Anopheles stephensi* midgut and promoted the growth of endogenous as well as exogenous fed bacteria. In addition, the mosquito midgut also efficiently regulated the number of these bacteria through the induction of classical Toll and Imd immune pathways. In case of AsHPX15 silenced midguts, the growth of midgut bacteria was largely reduced through the induction of nitric oxide synthase (NOS) gene, a downstream effector molecule of the JAK/STAT pathway. Interestingly, no significant induction of the classical immune pathways was observed in these midguts. Importantly, the NOS is a well known negative regulator of *Plasmodium* development, thus, we proposed that the induction of diverged immune pathways in the absence of HPX15 mediated midgut barrier might be one of the strategies to manipulate the vectorial capacity of *Anopheles* mosquito.

## Introduction

The tropical region of the world pays a heavy toll to malaria in terms of death and economic loss. World Health Organization reported 214 million cases and 438,000 deaths from malaria in 2015 (WHO, 2015). This situation certainly warrants attention to develop effective methods for malaria prevention. A number of strategies such as, discovery of effective vaccine/drug against malaria parasite *Plasmodium* and numerous vector control measures are worth mentioning here. However, the worldwide reporting of drug resistance in *Plasmodium* is also the major challenge in this field (Farooq and Mahajan, 2004).

Arresting *Plasmodium* development inside the mosquito host and developing transmission blocking strategies may also be promising in controlling malaria spread among the human hosts. *Plasmodium* completes its sexual life cycle in mosquito and asexual cycle continues in human host. Mosquito midgut is the first organ where mosquito-parasite interactions are initiated. In the midgut, *Plasmodium* undergoes a series of complex developmental transitions and majority of parasites are killed during this process (Ghosh et al., 2000; Sinden and Billingsley, 2001; Pradel, 2007). It is of note that the number of *Plasmodium* is regulated by the ingested blood factors as well as mosquito innate immunity (Lensen et al., 1998; Michel and Kafatos, 2005; Vlachou et al., 2005; Ramiro et al., 2011; Simon et al., 2013).

Several studies revealed that wild type or laboratory-reared mosquito midgut is housed by a variety of bacteria (Minard et al., 2013; Kajla et al., 2015a) and their interaction with different development stages of *Plasmodium* also determines the mosquito cycle of the parasite (Touré et al., 2000; Dong et al., 2009; Cirimotich et al., 2011b; Wang et al., 2011; Boissière et al., 2012; Moreno-García et al., 2014). In addition, the type and number of bacteria also vary during different development stages and habitat of the mosquito (Wang et al., 2011; Minard et al., 2013; Kajla et al., 2015a). Researchers exploited these findings to identify the mosquito gut-specific symbionts that can regulate *Plasmodium* development (Boissière et al., 2012). Gram-negative bacteria (for example, *Serratia marcescens, Enterobacter* species such as *E. amnigenus, E. cloacae, and E. sakazaki*) have been identified in anophelines midgut that inhibit *Plasmodium falciparum or P. vivax* sporogonic development. Mosquitoes treatment with antibiotics reduced the number of midgut bacteria that, in turn, reversed their effect on *Plasmodium* development (Pumpuni et al., 1993, 1996; Beier et al., 1994; Gonzalez-Ceron et al., 2003; Cirimotich et al., 2011a). Interestingly, in an artificial feeding, the co-infection of bacteria with *Plasmodium* in field collected or lab-reared mosquitoes also reduced the number of developing parasites (Dong et al., 2009; Cirimotich et al., 2011a). Thus, understanding the mechanisms behind these regulations will provide an excellent tool to develop *Plasmodium* control strategies. Earlier reports indicated that the bacteria suppressed *Plasmodium* infection through numerous mechanisms. For example, various enzymes and toxins produced by bacteria or the mosquito immunity or reactive oxygen species (ROS) induced against bacteria might kill the parasite (Pumpuni et al., 1993; Azambuja et al., 2005; Dong et al., 2009; Cirimotich et al., 2011a).

It is noteworthy to mention that the growth of midgut endogenous bacteria is induced extensively after the blood feeding. In addition, the feeding of exogenous bacteria supplemented blood also increases bacterial burden in the mosquito midgut. However, the midgut has a remarkable capacity to manage the midgut environment in a way to minimize the deleterious effects of the increased bacterial load (Warr et al., 2008; Dong et al., 2009; Gupta et al., 2009; Meister et al., 2009; Kajla et al., 2015a). Recent studies in *A. gambiae* mosquito identified a mechanism that modulates the innate immunity against bolus bacteria (Kumar et al., 2010; Kajla et al., 2015a). In these mosquitoes, a heme peroxidase AgHPX15 catalyzes the crosslinking of a mucin layer at the luminal surface of the midgut epithelium. This crosslinked mucin acts like a barrier to block the interaction of bolus bacteria or bacterial elicitors with the immune reactive midgut epithelium. This mechanism also provides a protected environment for the development of *Plasmodium* inside the midgut. Interestingly, AgHPX15 silencing resulted in induction of key antibacterial immune genes and decreased bacterial load in the midgut (Kumar et al., 2010; Kajla et al., 2015a). In addition, the reduction of *Plasmodium* development was also evident in these silenced midguts. It is also noteworthy to mention that HPX15 is a unique anopheline-lineage specific gene and its orthologs are absent in insects and human, however, they are present in the genome of nineteen worldwide distributed species of *Anopheles* mosquito and share 65–99% amino acids identity (Kajla et al., 2015b, 2016a).

These facts prompted us to develop a hypothesis that an increased load of exogenous bacteria in HPX15 silenced anopheline midguts will markedly induce the mosquito immunity due to the uninterrupted interactions of bacterial elicitors with the immune reactive midgut epithelium. Exploitation of this mechanism will be helpful to manipulate the vectorial capacity of the insect host. Thus, in the present study, we analyzed the mechanism of immunomodulation in the AsHPX15 silenced *Anopheles stephensi* midguts to understand the overview of microbial interactions with the mosquito defense system in this body compartment.

## Materials and Methods

### Rearing of Mosquitoes

*Anopheles stephensi* mosquitoes were reared in insectory at 28°C, 80% relative humidity (RH) and 12 h light:dark cycles as described before (Kajla et al., 2016b). Larvae were fed on a mixture of dog food (PetLovers Crunch), fish food (Toya), and tetramine (in 1:1:1 ratio). Adults were fed on 10% sucrose solution ad labitum. To maintain mosquito colony, adult females were also fed on anesthetized mice and the egg laid by them were collected in moist conditions. The use of animals in the study was approved by the Institutional animal ethics committee.

### Bacteria Feeding to the Mosquitoes and Collection of Tissues

Wild type *Escherichia coli* (MTCC 40) and *Micrococcus luteus* (MTCC 106) bacteria were obtained from the Microbial Type Culture Collection (MTCC), Institute of Microbial Technology (IMTECH), Chandigarh, India and used throughout the study. Bacteria were grown separately in LB media following standard protocols (Gupta et al., 2009). The growth of bacteria in culture was estimated by reading the absorbance at 600 nm. 1.5 ml of each bacterium culture (*A*_600_ = 0.5) was mixed and centrifuged at 2300 × *g* for 5 min. The cell pellet was washed twice with Ashburner’s PBS (3 mM Sodium chloride, 7 mM Disodium hydrogen phosphate, and 3 mM Sodium dihydrogen phosphate, pH 7.2) and finally suspended in 3 ml fresh mouse blood (10^9^ bacterial cells/ml blood). Overnight starved adult *A. stephensi* females were allowed to feed on mouse blood supplemented with or without bacterial suspension through an artificial membrane feeder as before (Dong et al., 2006; Gupta et al., 2009; Kumar et al., 2010). After different time of blood feeding the mosquito midguts were dissected, collected in RNAlater and stored at -80°C. Sugar fed midguts served as controls and were also collected in the same way.

### RNA Isolation, cDNA Synthesis, and Real Time PCR

Total RNA was isolated from the midgut tissues using the RNAeasy Mini kit (Qiagen, Cat no. 74104) and following manufacturer’s instructions. First-strand cDNA was synthesized using Quantitech reverse transcription kit (Qiagen, Cat no. 205311). The expression of various genes (as mentioned in the **Table 1**) was analyzed through qPCR using Sybr Green Master Mix (Biorad Cat no. 170-8882) and normalized against the constitutively expressed S7 ribosomal protein gene as before (Kajla et al., 2016a). The PCR was initiated with denaturation at 95°C for 3 min followed by 35 additional cycles at 94°C for 10 s, 57°C for 30 s and 72°C for 50 s. The final extension was carried at 72°C for 10 min and ^ΔΔ^Ct method was used to calculate the relative expressions of the target genes in the samples against the controls as discussed before (Livak and Schmittgen, 2001).

**Table 1:** The PCR primer sequences and their products with *A. stephensi* cDNA template.

S. No	Primers sets	Primer sequence (5′-3′)	PCR product (bp)	Reference
1.	HPX15 Fw2 HPX15 Rev2	GAGAAGCTTCGCACGAGATTA GAATGTCGATTGCTTTCAGGTC	329	Kajla et al., 2015b
2.	SOCS Fw SOCS Rev	CGTCGTACGTCGTATTGCTC CGGAAGTACAATCGGTCGTT	241	Dhawan et al., 2015
3.	NOS Fw NOS Rev	ACATCAAGACGGAAATGGTTG ACAGACGTAGATGTGGGCCTT	250	Present study
4.	GNBP Fw GNBP Rev	GAGTTCCAGTGGTACACCAACA CTTCGGCAGCAACCAGAT	333	Present study
5.	Toll Precursor Fw Toll Precursor Rev	ACCTGTCGGCGAATCCTTGG TCATCCTTGTGCGAGTACGA	358	Present study
6.	Gambicin Fw Gambicin Rev	GTGCTGCTCTGTACGGCAGCCG CTTGCAGTCCTCACAGCTATTGAT	344	Present study
7.	HPX8 Fw HPX8 Rev	GATCCTTTGCCGATGCGCTCAAT CAGTTCGGGCAGTTTATGTCGCAC	318	Present study
8.	16S Fw 16S Rev	TCCTACGGGAGGCAGCAGT GGACTACCAGGGTATCTAATCCTGTT	467	Kumar et al., 2010
9.	S7 Fw S7 Rev	GGTGTTCGGTTCCAAGGTGA GGTGGTCTGCTGGTTCTTATCC	487	Vijay et al., 2011

### dsRNA Synthesis and Gene Silencing

LacZ (218bp) or AsHPX15 (428bp) was cloned in TOPO 2.1 vector and PCR amplified using M13F, 5′GTAAAACG ACGGCCAGT3′ and T7-M13R, 5′CTCGAGTAATACGAC TCACTATAGGGCAGGAAACAGCTATGAC3′ primers with the following conditions; 94°C for 5 min, 40 cycles of 94°C for 30 s, 55°C for 30 s, 72°C for 30 s, and final extension was carried at 72°C for 10 min. Amplicons were purified with the QIAquick PCR Purification Kit (Qiagen, Cat No 28104). PCR-purified amplicons tailed with T7 promoter sequences were used to synthesize dsRNA with the MEGAscript kit (Ambion) following the manufacturer’s instructions. For dsRNA mediated interference (RNAi), 1–2 days old female mosquitoes were injected with 69 nl of 3 μg/μl dsLacZ (controls) or dsAsHPX15 (silenced) RNA into their thoraxes using a nanojector (Drummond, Broomall, PA, USA). Five days after the injection, mosquitoes were allowed to feed on mouse blood supplemented with or without 10^9^ bacterial cells/ml as discussed above. Tissue collection and gene specific PCR was carried as mentioned above.

### Statistical Analysis of the Data

All the data were expressed as mean ± standard deviation. Statistical significance between test and respective controls was analyzed by *t*-test using GraphPad Prism 5.0 software (Motulsky, 1999). The data with *P* < 0.05 was considered significant.

## Results

### Growth Pattern of Exogenous Fed Bacteria in Mosquito Midgut

**FIGURE 1:**
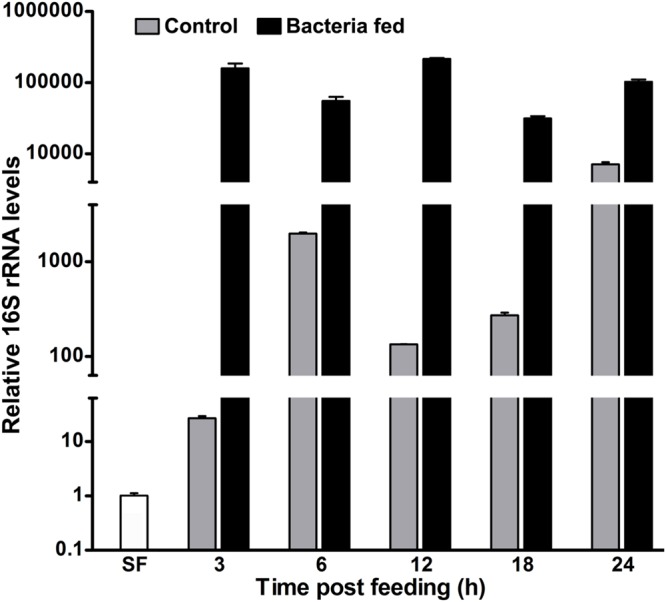
**The kinetics of 16S rRNA levels in blood fed mosquito midguts.** Mosquitoes were fed either on blood alone (controls) or supplemented with a mixture of *M. luteus* and *E. coli* bacteria (10^9^ cells/ml blood). The relative levels of 16S rRNA were analyzed in the pool of mosquito midguts collected at different time intervals after the feeding and represented here in log_10_ scale. SF represents the 16S rRNA levels of sugar fed midguts.

Mosquito midgut is housed by a diverged group of naturally acquired (endogenous) bacteria. The majority of these bacteria are Gram-negative and their growth is induced after blood feeding (Rani et al., 2009; Wang et al., 2011; Boissière et al., 2012; Chavshin et al., 2012). In parallel, the blood feeding also induces the formation of peritrophic matrix, an acellular barrier that separates blood bolus from the midgut epithelium (Kumar et al., 2010; Injera et al., 2013). Additionally, a heme peroxidase HPX15 crosslinked mucin barrier on the luminal surface of the epithelium also help to maintain a low immunity zone in the blood fed midgut to support the growth of endogenous bacteria (Kumar et al., 2010; Kajla et al., 2015a). We were interested to understand the relationship between the growth of exogenous fed bacteria and midgut immune responses in the presence of *A. stephensi* AsHPX15 crosslinked mucin barrier. For that, the adult females were fed on blood supplemented without or with a mixture of Gram^+^ (*M. luteus*) and Gram^-^ (*E. coli*) bacteria and the growth kinetics of midgut bacteria was analyzed. Results presented in **Figure 1** revealed that the relative levels of 16S rRNA for endogenous bacteria increased 25-fold after 3 h of blood feeding when compared to the sugar fed midguts (*p* = 0.010). Afterward, the relative levels of endogenous bacteria were 1900, 133, and 270 times after 6, 12, and 18 h of post blood feeding, respectively, against the sugar fed controls (**Figure 1**). This showed significant reduction in the levels of endogenous bacteria at 12 h (*p* = 0.0008) and 18 h (*p* = 0.0011) when compared to the 6 h blood fed midguts. Furthermore, the endogenous bacteria levels increased 7000 times in 24 h blood fed midguts in comparison to the sugar fed midguts (**Figure 1**). These results are in agreement with previous findings where an increase in endogenous bacteria was observed after 6 h of blood feeding and that further followed the similar growth pattern (Luckhart et al., 1998; Kumar et al., 2010).

Parallelly, in case of exogenous bacteria fed midguts, the relative levels of 16S rRNA were 150,000-fold at 3 h in comparison to the sugar fed midguts (**Figure 1**). Interestingly, the relative 16S rRNA levels were indifferent at 6 h (*p* = 0.0645) and 12 h (*p* = 0.1696), respectively, when compared to 3 h post bacteria fed midguts. However, the expression levels of 16S rRNA gene at 18 h were reduced fivefold (*p* = 0.0041) against 3 h post fed midguts (**Figure 1**). Thus, these altered levels of 16S rRNA at different time points indicated that the growth of bacteria is regulated rhythmically, most probably by the midgut immunity.

It is noteworthy to mention that in our study, we fed a large amount of bacteria (10^9^ bacterial cells/ml blood) to the mosquitoes. The comparative levels of 16S rRNA revealed that the exogenous bacteria fed midguts have 28- and 15-fold higher bacteria load at 6 and 24 h, respectively, when compared to the just blood fed midguts (**Figure 1**). The reduction in the folds ratio at 24 h seems to be due to the decreased levels of 16S rRNA in exogenous bacteria fed midguts and increased levels of endogenous bacteria in the corresponding blood fed controls. This might indicate that the midgut environment is particularly favorable for the growth of endogenous bacteria in comparison to the exogenous fed microbes.

### AsHPX15 Gene is Induced in Exogenous Bacteria Fed Midguts

**FIGURE 2:**
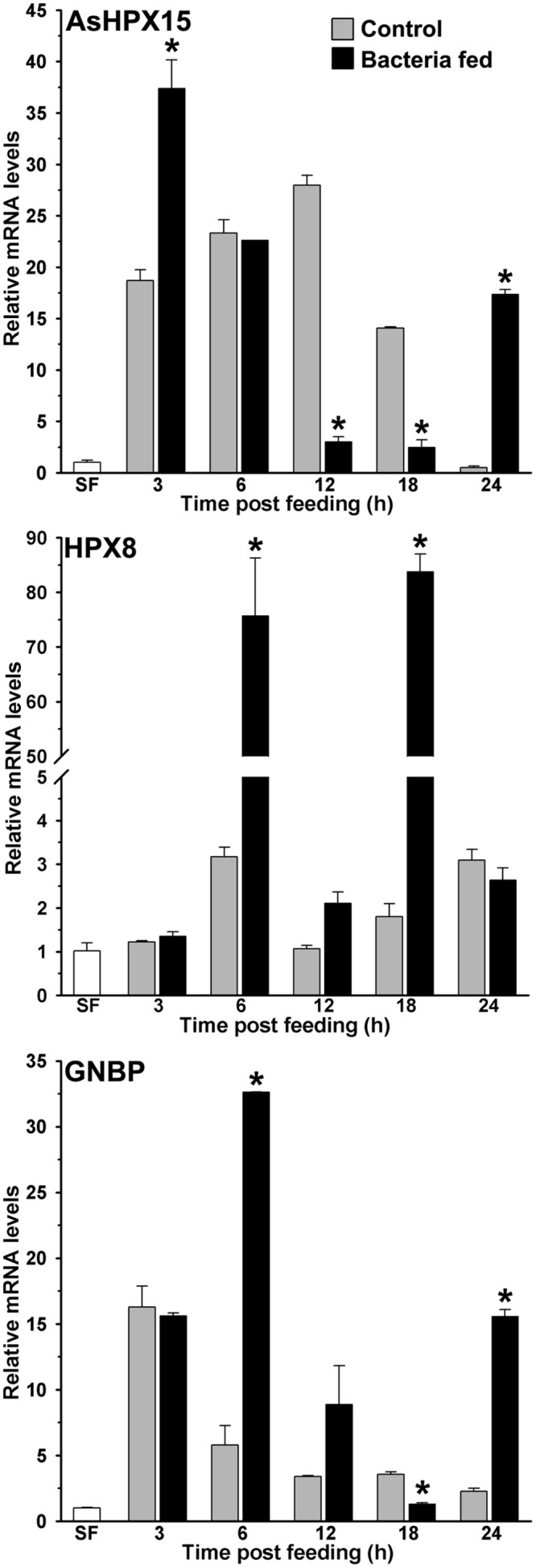
**Expression kinetics of heme peroxidases and pattern recognition receptor (PRR) in bacteria fed midguts.** The kinetics of relative mRNA levels of AsHPX15, HPX8, or GNBP gene were analyzed in the blood alone (controls) or supplemented with a mixture of *M. luteus* and *E. coli* bacteria fed midguts. SF represents the mRNA levels of these genes in sugar fed midguts. Significant differences in the gene expressions between control and bacteria fed midguts are denoted by asterisks.

Previously, we found that the heme peroxidase HPX15 gene is highly induced in *A. gambiae* or *A. stephensi* blood fed midguts (Kumar et al., 2004; Kajla et al., 2016a). This molecule is important to suppress the immune activation and facilitating the growth of endogenous bacteria in the blood fed midguts (Kumar et al., 2010; Kajla et al., 2015a). We also analyzed the expression kinetics of AsHPX15 gene in those midguts that are mentioned in **Figure 1**. We found that AsHPX15 gene was induced 23- and 27-fold at 6 and 12 h post blood feeding, respectively, when compared to the sugar fed midguts (**Figure 2**). The induction of AsHPX15 gene in 6 h post bacteria supplemented blood fed midguts was similar to the blood fed controls (*p* = 0.6485). However, its expression levels were reduced drastically at 12 and 18 h in bacteria fed midguts against blood fed controls (*p* = 0.0018 for 12 h and *p* = 0.0038 for 18 h**)**. Interestingly, the relative AsHPX15 mRNA levels were higher in 3 and 24 h post bacteria fed midguts against the blood fed controls. Thus, the heavy load of exogenous bacteria in the midgut had no effect on the early phase of AsHPX15 gene expression (**Figure 2**). This may be simply due to the regulation of this gene by ecdysone hormone, which is induced in blood fed mosquitoes as reported before (Kajla et al., 2016a). We also believed that AsHPX15 catalyzed mucin barrier might be completed or its formation started within the first 12 h of post blood feeding because the presence of mucin and AgHPX15 protein is detected at the luminal surface of *A. gambiae* midgut epithelium by this time (Kumar et al., 2010; Injera et al., 2013). On the other hand, if the mucin barrier formation is completed and it suppresses the midgut antibacterial immunity, then the over growth of exogenous bacteria might be lethal for the mosquito. However, we found that the mortality in blood fed controls and exogenous bacteria fed mosquitoes was indifferent. This indicated that mosquito gut immunity is capable of counteracting the increased load of bacteria without compromising its survival.

### The Antibacterial Heme Peroxidase HPX8 is Induced in Exogenous Bacteria Fed Midguts

Our results revealed that the numbers of exogenously fed bacteria not only increase in the mosquito midgut, it is also regulated at different time points (**Figure 1**). Thus, we hypothesized that some of the immune genes might be controlling the overgrowth of bacteria in these midguts. To test this concept, we analyzed the expression of a heme peroxidase HPX8, which has been reported to be an antibacterial gene in *A. gambiae* (Kumar et al., 2010). Results presented in **Figure 2** revealed that HPX8 mRNA is induced 25- and 42-fold at 6 and 18 h, respectively, in bacteria fed midguts against the blood fed controls (*p* = 0.0206 for 6 h and *p* = 0.0015 for 18 h), however, at other time points, HPX8 mRNA levels in the test and controls were indifferent. This induction pattern of HPX8 gene (**Figure 2**) corroborated with the bacterial 16S rRNA levels in these midguts (**Figure 1**). These findings indicated that HPX8 might be one of the candidate genes that participate in the midgut antibacterial immunity.

### Classical Immune Pathways are Induced in Exogenous Bacteria Fed Midguts

In insects, Gram-negative binding protein (GNBP) acts like the pattern recognition receptor (PRR) and triggers classical innate immunity through Toll or Imd pathway against Gram-positive or Gram-negative bacteria, respectively (Lemaitre et al., 1995; Michel et al., 2001; Osta et al., 2004; Waterhouse et al., 2007). In anopheline mosquitoes, several isoforms of GNBP have been identified (Dimopoulos et al., 1998; Christophides et al., 2002; Osta et al., 2004; Warr et al., 2008) thus, we designed a set of common primers to detect the expression of any given isoform of GNBP in our study (primer sequences are provided in **Table 1**). Our analyses revealed that mRNA levels of GNBP were induced ∼17-fold after 3 h of blood feeding when compared to the sugar fed midguts and that remained unaffected by the presence of bacteria in the blood (**Figure 2**). However, at 6 and 12 h post bacteria feeding, the relative mRNA levels of GNBP were six and two folds higher, respectively, against the normal blood fed controls (**Figure 2**). Furthermore, the relative mRNA levels of GNBP were seven folds higher in 24 h post bacteria fed midguts in comparison to the blood fed controls (**Figure 2**, *p =* 0.0019). These findings indicated that GNBP induction represents two episodes, an early event around 6 h and a late phase expression around 24 h post bacteria feeding. Thus, to further explore the immune regulatory mechanism that balances the mosquito midgut immunity against exogenous bacteria, these two time points were further analyzed in detail.

**FIGURE 3:**
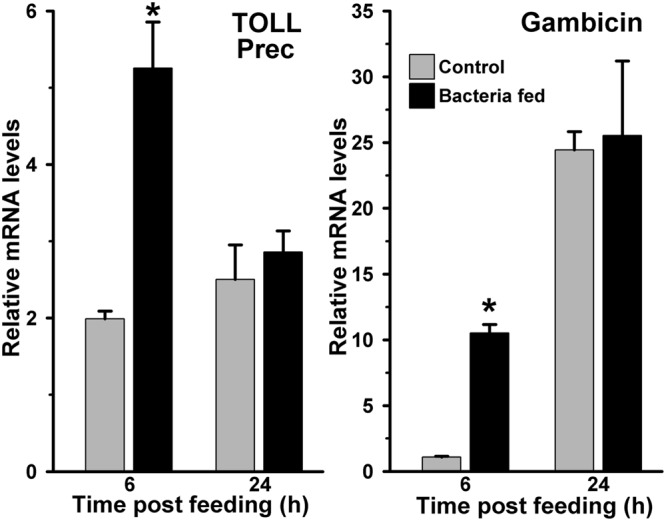
**Expression of Toll and gambicin immune genes in bacteria fed *Anopheles stephensi* midguts.** mRNA levels of midgut Toll (Toll precursor) or gambicin gene were analyzed at 6 and 24 h after feeding the blood alone (controls) or supplemented with a mixture of *E. coli* and *M. luteus*. Relative levels of mRNA are presented against the sugar fed midguts. Significant differences in the gene expression levels are denoted by an asterisk.

We studied the induction of Toll and Imd pathways in the above-mentioned samples those were collected at early 6 h and late 24 h post feeding. The gene expression analyses revealed that the Toll mRNA levels were ∼three folds higher in the 6 h post bacteria fed midguts in comparison to the blood fed controls (**Figure 3**, *p* = 0.036). However, the induction of this gene was indifferent at 24 h in these midguts (*p* = 0.563). In addition, we also analyzed the expression of gambicin in these samples. Gambicin is an effector antimicrobial peptide, which is regulated by both Toll and Imd pathways (Vizioli et al., 2001). The results presented in **Figure 3** revealed that gambicin was induced 10-folds higher in 6 h post bacteria fed midguts when compared to the blood fed controls (*p* = 0.004). Although, gambicin expression is further induced in 24 h post bacteria fed midguts, however, these levels were similar to the blood fed controls (*p* = 0.294). Because gambicin is effective against both Gram^+^ and Gram^-^ bacteria, therefore, its expression at early and late time points corroborate with the increased number of midgut bacteria (compare **Figures 1** and **3**). In conclusion, the induction of classical Toll and Imd immune pathways regulate the growth of bacteria in blood fed midguts.

### JAK/STAT Pathway is Not Induced in Exogenous Bacteria Fed Midguts

**FIGURE 4:**
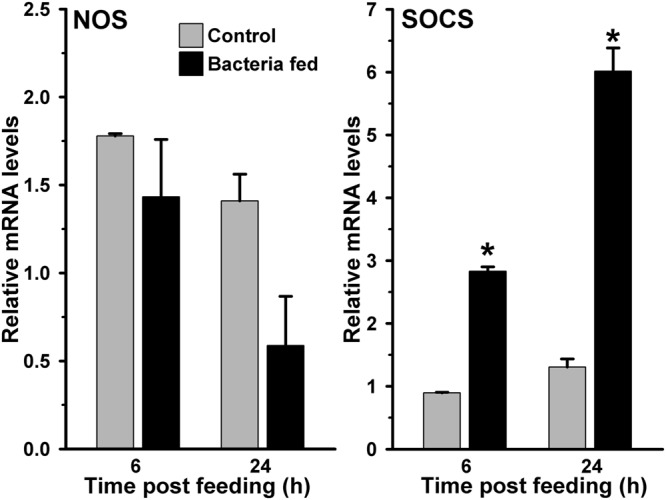
**Expression of NOS and SOCS immune genes in bacteria fed *A. stephensi* midguts.** Midgut mRNA levels of NOS or SOCS gene were analyzed at 6 and 24 h after feeding the blood alone (controls) or supplemented with a mixture of *E. coli* and *M. luteus*. Relative levels of mRNA are presented against the sugar fed midguts. Significant differences in the gene expression levels are shown by asterisks.

Another mosquito immune pathway, known as JAK/STAT, is also reported to be involved in gut immunity against a variety of pathogens (Gupta et al., 2009; Kumar et al., 2010). In this pathway, the receptor mediated activation of tyrosine kinases (JAKs) initiates the phosphorylation of cytosolic STAT (Signal transducer and activator of transcription) proteins. The phosphorylated STATs make dimer and upon translocation into the nucleus, they regulate the expression of numerous immune genes. One of such STAT-mediated effector gene nitric oxide synthase (NOS) is reported to play a crucial role in controlling the pathogenic development in *Anopheles* mosquito (Gupta et al., 2009; Kumar et al., 2010; Bahia et al., 2011). We were also interested to understand the activation of STAT pathway in exogenous bacteria fed midguts. Results presented in **Figure 4** revealed that the NOS mRNA levels in 6 and 24 h post bacteria fed midguts were indifferent from the blood fed midguts (*p* = 0.399 at 6 h and *p* = 0.123 at 24 h). Collectively, these results suggested that NOS is not induced in the midgut against exogenous fed bacteria. Thus, we believed that the STAT pathway does not participate in *A. stephensi* midgut antibacterial immunity and confirms the previous findings as reported in other mosquitoes (Agaisse and Perrimon, 2004; Gupta et al., 2009; Kumar et al., 2010).

We hypothesized that either the STAT pathway in above-mentioned midguts remain uninduced or it is suppressed by the bacteria through some specific mechanism. For that, we analyzed the expression of the suppressor of cytokine signaling (SOCS) gene, which is a downstream feedback repressor of the STAT pathway (Wormald and Hilton, 2004; Gupta et al., 2009; Dhawan et al., 2015). Interestingly, the SOCS is induced ∼four and ∼five folds at 6 and 24 h, respectively, in the bacteria fed midguts against blood fed controls (**Figure 4**, *p* = 0.001 for 6 h and *p* = 0.006 for 24 h). These results revealed that the induction of SOCS gene in bacteria fed midguts might be responsible for inhibiting the activation of STAT pathway and NOS expression in a way similar to other systems (Zhang et al., 2011; Demirel et al., 2013; Maehr et al., 2014).

### AsHPX15 Silencing Suppressed the Growth of Exogenous Bacteria in the Midgut

**FIGURE 5:**
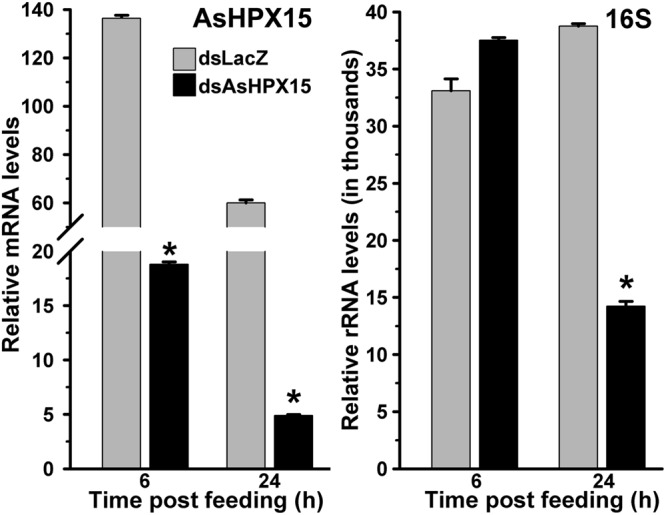
**Relative levels of AsHPX15 mRNA and 16S rRNA in AsHPX15 silenced and bacteria fed midguts.** Mosquitoes injected with dsLacZ (controls) or dsAsHPX15 (silenced) RNA were fed on bacteria supplemented blood and relative levels of AsHPX15 mRNA and 16S rRNA were analyzed in their midguts at different time intervals after feeding. Relative mRNA levels are presented against the sugar fed midguts. Significant differences are shown by asterisks.

We were interested to understand the mosquito midgut immune regulation against high load of the exogenous bacteria when HPX15 crosslinked mucin barrier formation is suppressed. For that, we silenced AsHPX15 gene through dsRNA-mediated interference as discussed in Materials and Methods. Silenced mosquitoes were fed on blood supplemented with bacteria (a mixture of *M. luteus* and *E. coli*). The midguts were collected at early 6 h and late 24 h post feeding to analyze the expression profiling of the immune genes as mentioned above. Our analysis of AsHPX15 mRNA levels in controls and silenced midguts revealed that this gene was silenced in the range of 80–90% (**Figure 5**). The relative 16S rRNA levels in controls and silenced midguts were similar at 6 h post feeding (*p* = 0.053). However, at 24 h post feeding there was 63% reduction in the 16S rRNA levels in the silenced midguts against the unsilenced controls (**Figure 5**, *p* = 0.0004). Thus, AsHPX15 silencing reduces the overall growth of bacteria in the midgut bolus that might include the endogenous as well as exogenous bacteria. These findings are in partial agreement with previous reports where silencing of *A. stephensi* AsHPX15 ortholog drastically reduced the growth of endogenous bacteria in *A. gambiae* midguts (Kumar et al., 2010; Kajla et al., 2015a).

### Classical Antibacterial Immune Genes are Suppressed in AsHPX15 Silenced Midguts

**FIGURE 6:**
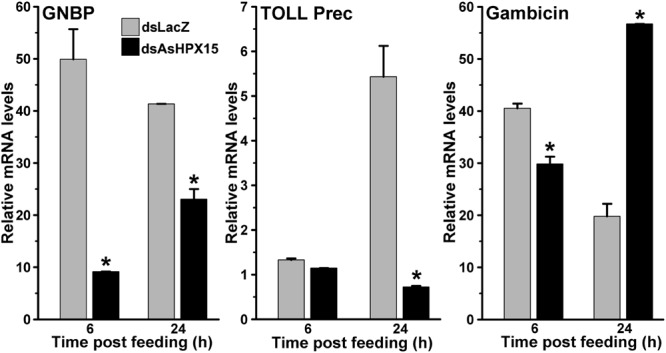
**Expression of classical immune pathway genes in AsHPX15 silenced and bacteria fed midguts.** Mosquitoes injected with dsLacZ (controls) or dsAsHPX15 (silenced) RNA were fed on bacteria supplemented blood and relative mRNA levels of GNBP, Toll (Toll precursor) or gambicin gene were analyzed in their midguts after 6 and 24 h post feeding. Relative levels of mRNA are presented against the sugar fed midguts. Significant differences between dsLacZ and dsAsHPX15 samples are shown by an asterisk.

To understand the immune regulation of bacteria in the AsHPX15 silenced midguts, we analyzed the expression profile of important classical immune pathways. Results presented in **Figure 6** revealed that the mRNA levels of the PRR GNBP were reduced significantly in silenced midguts at 6 h (*p* = 0.005) and 24 h (*p* = 0.0111) post bacteria feeding, when compared to their respective unsilenced controls. Thus, the reduced levels of GNBP expression in silenced midguts might reflect the compromised situation for the recognition and induction of classical immune pathways. This fact was further confirmed after analyzing the gene expression of Toll and Imd pathways in these samples. We found that the levels of Toll mRNA were indifferent in silenced and control midguts after 6 h of bacteria feeding (**Figure 6**, *p* = 0.123). However, Toll mRNA levels were suppressed seven folds in the silenced midguts than controls at 24 h post bacteria feeding (*p* = 0.0209). These results revealed that Toll pathway is not induced in AsHPX15 silenced midguts neither at early nor at late stage of post bacteria feeding. Furthermore, we analyzed the expression of gambicin in these AsHPX15 silenced samples. We observed that the expression of gambicin gene was significantly downregulated in the silenced midguts after 6 h post feeding against controls (**Figure 6**, *p* = 0.023). Interestingly, at 24 h post feeding the expression of gambicin gene was induced ∼three folds in silenced midguts in comparison to the controls (*p* = 0.004). These findings suggested that the induction of gambicin in 24 h silenced midguts might be regulated by some other mechanism(s) that is different from classical Toll and Imd pathways.

### JAK/STAT Pathway is Induced against Exogenous Bacteria in AsHPX15 Silenced Midguts

**FIGURE 7:**
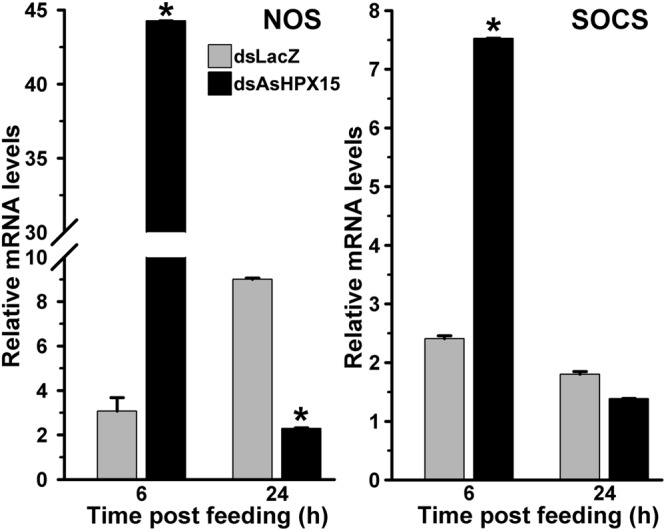
**Relative levels of NOS and SOCS mRNA in AsHPX15 silenced and bacteria fed midguts.** Mosquitoes injected with dsLacZ (controls) or dsAsHPX15 (silenced) RNA were fed on bacteria supplemented blood and relative levels of NOS or SOCS mRNA were analyzed in their midguts at different time intervals after feeding. Relative levels of mRNA are presented against the sugar fed midguts. Significant differences are denoted by an asterisk.

We further examined the JAK/STAT pathway genes in AsHPX15 silenced midguts to understand the involvement of this pathway in antibacterial immunity. For that, we compared the expression of STAT pathway genes in silenced or sham treated mosquito midguts after bacteria supplemented blood feeding. Results presented in **Figure 7** revealed that NOS and SOCS genes were induced 15- and 3-folds, respectively, in the silenced midguts after 6 h of bacteria feeding when compared to the controls (*p* = 0.0002 for NOS and *p* < 0.0001 for SOCS expression). However, the expressions of both these genes in the silenced midguts were reduced 4- and 1.3-folds, respectively, after 24 h post feeding against unsilenced controls (*p* < 0.0001 for NOS and *p* = 0.068 for SOCS expression). This pattern of NOS and SOCS expression indicated the activation of STAT pathway in silenced midguts. Interestingly, the profile of NOS expression also corroborated with the levels of bacteria in the silenced midguts (Comparing **Figures 5** and **7**). We believed that the induced NOS during the early hours (6 h, **Figure 7**) might be responsible for reducing the levels of bacteria at later time points (24 h, **Figure 5**) as this gene is reported to play an antibacterial role in mosquitoes as well as other insects (Hillyer and Estévez-Lao, 2010). These results collectively revealed that STAT pathway is actively engaged in antibacterial immunity in the AsHPX15 silenced *A. stephensi* midguts. This effect might be due to the reduction of AsHPX15 catalyzed mucin barrier crosslinking and that, in turn, allows direct interaction of bacteria or bacterial elicitors with immune reactive midgut epithelium.

### The Antibacterial Peroxidase HPX8 is Suppressed in AsHPX15 Silenced Midguts

**FIGURE 8:**
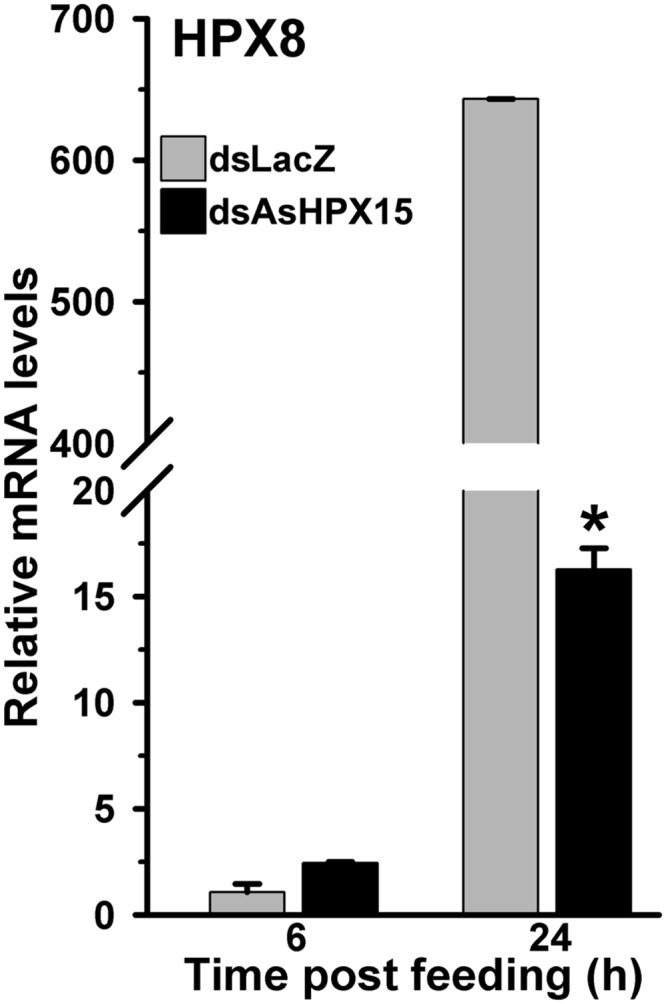
**Expression of HPX8 gene in AsHPX15 silenced and bacteria fed midguts.** Mosquitoes injected with dsLacZ (controls) or dsAsHPX15 (silenced) RNA were fed on bacteria supplemented blood and relative levels of HPX8 mRNA in their midguts were analyzed at different time points after feeding. Relative levels of mRNA are presented against the sugar fed midguts. Significant differences are shown by an asterisk.

As, we found before that HPX8, an antibacterial peroxidase, is induced after the exogenous bacteria feeding (**Figure 2**). We analyzed the expression of this gene to understand its role in regulating the bacterial load when AsHPX15 mediated physical barrier formation is interrupted. Results presented in **Figure 8** revealed that in dsLacZ injected control midguts, HPX8 gene was induced 650-fold after 24 h of exogenous bacteria feeding against sugar fed midguts. However, its expression was reduced ∼40 times in AsHPX15 silenced midguts when compared to the unsilenced controls (**Figure 8**). Moreover, the expression levels of HPX8 gene in controls and silenced midguts at 6 h post feeding were indifferent (*p* = 0.078).

These results collectively indicated that the classical antibacterial pathways are least effective in controlling the heavy load of exogenous bacteria in AsHPX15 silenced midguts. However, the induced NOS (an effector gene of the JAK/STAT pathway) and gambicin seem to be the major determinants of the antibacterial immunity in these midguts.

## Discussion

The mosquito midgut is an organ for food digestion and immunity. It is an excellent system to understand the interaction of innate immunity, exogenous food and endogenous natural microbial habitats. It is noteworthy to mention that a fine balance between the innate immunity and digestion process is required to drive the physiological functions of the midgut over the immune reactivity against bolus antigens. For that, mosquito midgut is equipped with a number of mechanisms (Kajla et al., 2015a). Among these mechanisms, the formation of a heme peroxidase HPX15 crosslinked mucin barrier is extremely important (Kumar et al., 2010). This mucin barrier does not allow the interaction and recognition of bolus bacteria with the immune reactive midgut epithelium and thus, protects them from the immune attack. As a result of that food digestion or other important physiological processes are driven forward without the induction of midgut immunity (Kajla et al., 2015a). Our data also supported this concept that the endogenous as well as exogenous fed bacteria easily grow in the midgut lumen and AsHPX15 gene is induced after feeding the normal or bacteria supplemented blood (**Figures 1** and **2**). Interestingly, we observed the reduction of AsHPX15 gene expression in 12 h post bacteria fed midguts (**Figure 2**). This effect might be due to the SOCS mediated suppression of STAT pathway (**Figure 4**). The presence of STAT binding site in the regulatory region of AsHPX15 gene supported this assumption (Kajla et al., 2016a). We also believed that the suppression of AsHPX15 gene expression in bacteria fed midguts might be least effective on the mucin barrier formation because the luminal secretion of mucins and localization of HPX15 protein occur by this time, which is well evident in the case of *A. gambiae* midguts (Kumar et al., 2010; Injera et al., 2013).

Importantly, the number of midgut bacteria does not increase continuously; however, there is variability in their growth pattern (**Figure 1**). It may be due to the selection and growth of specific types of midgut bacteria at certain time points or all types of bacteria have variable growth patterns at different time of post blood feeding. Moreover, these assumptions warrant further investigations. On the other hand, the midgut immunity might also play an important role in regulating the growth of bacteria at certain moments therefore, some of the classical immune genes such as, HPX8, GNBP, Toll and gambicin are induced at 6 h after bacteria supplemented blood feeding (**Figures 2** and **3**). However, in case of bacteria-free blood fed control midguts, the induction of these genes is comparatively minimal to facilitate the growth of endogenous bacteria. Thus, the mosquito midgut system might determine the degree and strength of immune activation on the basis of bacterial or antigenic load in blood bolus. These findings may also explain the previous observations by other researchers where feeding the exogenous bacteria with *Plasmodium* infected blood reduced the parasite development (Pumpuni et al., 1993, 1996; Gonzalez-Ceron et al., 2003; Dong et al., 2009). We believed that the negative regulation of *Plasmodium* development in the presence of exogenous bacteria might be due to an early induction of midgut immunity as we observed in **Figure 7** because some of these immune pathways are also known to participate in antiplasmodial immunity (Cirimotich et al., 2011a; Bahia et al., 2014).

Our previous findings revealed that the anopheline heme peroxidase HPX15 is a unique lineage-specific gene and its role in the modulation of mosquito midgut immunity can be targeted to control *Plasmodium* development (Kajla et al., 2014, 2015b, 2016a). Thus, we thought to combine our present knowledge of exogenous bacteria induced immunity with AsHPX15 gene silencing to understand the additive effect of these two events on mosquito midgut immunity. We found that AsHPX15 silencing induced innate immunity to suppress the bacterial load in the silenced midguts and capable of regulating the high load of exogenous fed bacteria (**Figure 5**). These findings are in agreement with the previous study where silencing of AsHPX15 ortholog in *A. gambiae* suppressed the growth of endogenous bacteria through the induction of bacteria-specific classical immunity (Kumar et al., 2010). These effects are simply due to the disruption of mucin barrier in AsHPX15 silenced midguts and that leads to the recognition of the bolus bacteria by the immune reactive midgut epithelial cells (Kumar et al., 2010).

We observed that the regulation of exogenous fed bacteria in the mosquito midgut is due to the activation of classical Toll and Imd immune pathways when AsHPX15 mediated mucin barrier is present (**Figures 2** and **3**). The silencing of AsHPX15 gene and thus, reducing the formation of mucin barrier, might alter the number of bacteria in the midgut. In AsHPX15 silenced midguts the classical immune pathways are mostly ineffective (**Figure 6**) and the bacteria levels are regulated by other stronger immune mechanisms. Thus, NOS is activated at early hours in AsHPX15 silenced midguts to control the heavy load of bacteria (that includes exogenous as well as endogenous bacteria) (**Figure 7**). In addition, the induction of SOCS gene in parallel to the NOS might again indicate the activation as well as balanced regulation of JAK/STAT pathway in AsHPX15 silenced midguts (**Figure 7**). Our findings also revealed that the levels of endogenous bacteria are very less in comparison to the exogenous fed bacteria (**Figure 1**) thus, NOS is induced in AsHPX15 silenced midguts to control the outsized load of these bacteria (**Figure 7**). In addition, the induction of gambicin, a classical immune pathway gene, in AsHPX15 silenced midguts might indicate its regulation through JAK/STAT pathway as suggested by other researchers (Buchon et al., 2009; Cheng et al., 2016).

**FIGURE 9:**
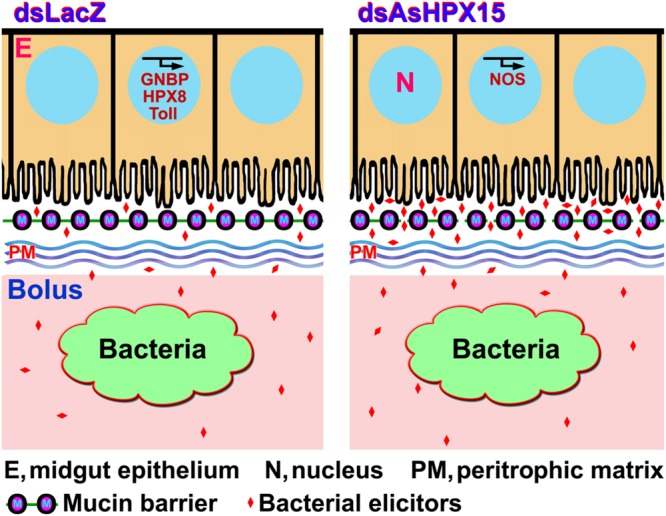
**Model of *A. stephensi* midgut immunity in the presence or absence of AsHPX15 gene.** AsHPX15 catalyzes the formation of mucin layer that blocks the direct interaction of lumen bacteria or bacterial elicitors with midgut epithelium. The increased load of bacteria induces classical immune pathways such as, GNBP, Toll and an antibacterial heme peroxidase HPX8 to regulate bacterial numbers. AsHPX15 silencing reduces the formation of mucin barrier that allows direct interaction of lumen bacteria or their elicitors with midgut epithelium. In this condition, STAT pathway effector molecule NOS is induced to regulate the numbers of midgut bacteria.

Immune regulation of a huge exogenous bacterial load in the AsHPX15 gene silenced midguts revealed that there is a circumstantial activation of antibacterial immunity. In other words, classical immune pathways are most active against increasing load of bacteria when AsHPX15 gene is expressed (**Figures 2** and **3**), however, STAT pathway predominates as antibacterial in the absence of AsHPX15 gene expression (**Figure 7**). This might indicate that the bacterial load and its interaction with the midgut epithelium boosts more effective immune molecule to manage the microbial homeostasis. Thus, we proposed a model as shown in **Figure 9** that AsHPX15 helps in the formation of mucin barrier in *A. stephensi* midgut. This mucin barrier blocks the direct interactions of bolus bacteria with the immune reactive gut epithelial cells. However, the increased growth of bacteria or their elicitors activate the classical Toll and Imd pathways that maintain gut bacterial homeostasis. On the other hand, in AsHPX15 silenced midguts the formation of mucin barrier is compromised and bacterial elicitors activate the midgut epithelial immunity. As a result, midgut epithelial cells mount a strong immune response through the activation of STAT pathway to regulate the growth of midgut bacteria and protecting the mosquito against its deleterious effects (**Figure 9**).

Based on our findings, we emphasized that manipulation of AsHPX15 gene in *A. stephensi* modulates the immune system and co-infection of *Plasmodium* with exogenous bacteria will result in the negative regulation of the parasite development through induction of specific immune pathways. Because HPX15 is highly conserved gene exclusively among anophelines (Kajla et al., 2015b, 2016a), then the proposed idea might be applicable for other worldwide distributed malaria vectors as a common strategy.

## Author Contributions

MK, LG, and SK designed experiments for this work. MK, TC, PK, KG, and RD collected samples to perform experiments. MK, TC, PK, KG, RD, LG, and SK performed AsHPX15 and bacterial kinetics experiments and analyzed the induction of different immune genes. MK, TC, PK, LG, and SK performed AsHPX15 silencing experiments. MK, LG, and SK analyzed the data and wrote the manuscript with input from all authors. All authors read and approved the manuscript.

## Conflict of Interest Statement

The authors declare that the research was conducted in the absence of any commercial or financial relationships that could be construed as a potential conflict of interest.
